# Validation of the self-report quantified Tuberous Sclerosis Complex-Associated Neuropsychiatric Disorders Checklist (TAND-SQ)

**DOI:** 10.1186/s13023-025-03642-2

**Published:** 2025-06-13

**Authors:** Nola Chambers, Tosca-Marie Heunis, Sugnet Gardner-Lubbe, Jamie K. Capal, Stacey Bissell, Anna W. Byars, Sebastián Cukier, Peter E. Davis, Jennifer Flinn, Tanjala T. Gipson, J. Chris Kingswood, Aubrey J. Kumm, Eva Schoeters, Catherine Smith, Shoba Srivastava, Megumi Takei, Stephanie Vanclooster, Agnies M. van Eeghen, Robert Waltereit, Darcy A. Krueger, Mustafa Sahin, Liesbeth De Waele, Anna C. Jansen, Petrus J. de Vries

**Affiliations:** 1 Centre for Autism Research in Africa (CARA), Division of Child and Adolescent Psychiatry, University of Cape Town, 46 Sawkins Road, Rondebosch, Cape Town, 7700 South Africa; 2 Mental Health and Wellbeing Research Group, Department of Public Health, Vrije Universiteit Brussel, Brussels, Belgium; 3 Department of Statistics and Actuarial Sciences, MuViSU (Centre for Multi-Dimensional Data Visualisation), Stellenbosch University, Stellenbosch, South Africa; 4 Department of Neurology, University of Rochester Medical Center, Rochester, NY USA; 5 School of Psychology, University of Birmingham, Birmingham, UK; 6 Division of Neurology, Department of Pediatrics, Cincinnati Children’S Hospital Medical Center/University of Cincinnati College of Medicine, Cincinnati, OH USA; 7 Department of Psychopathology and Mental Health, Pedro de Elizalde Hospital, Buenos Aires, Argentina; 8 Department of Neurology, Boston Children’s Hospital and Harvard Medical School, Boston, MA USA; 9 TSC Canada, Mississauga, ON Canada; 10 Department of Pediatrics, The University of Tennessee Health Sciences Center, Memphis, TN USA; 11 Le Bonheur Children’s Hospital and The Boling Center for Developmental Disabilities, Memphis, TN USA; 12 Department of Clinical Genetics, St George’s University Hospitals, London, UK; 13 Sussex Renal Unit, The Royal Sussex County Hospital, Brighton, UK; 14 Be-TSC, Mortsel, Belgium; 15 TSCi, Mortsel, Belgium; 16 TSC Alliance, Silver Spring, MD USA; 17 Society of Parents of Children with Autistic Disorders (SOPAN), Maharashtra, India; 18 Japanese Society of Tuberous Sclerosis Complex, Family Network, Tokyo, Japan; 19 Emma Children’S Hospital, Amsterdam University Medical Centers, Amsterdam, The Netherlands; 20 TAND Expert Centre, ‘sHeeren Loo, Noordwijk, The Netherlands; 21 Department of Psychiatry, Psychotherapy and Preventive Medicine, Ruhr University Bochum, Bochum, Germany; 22 Child and Adolescent Psychiatry, LWL-Klinikum Marsberg, Marsberg, Germany; 23 TSC Clinic, Cincinnati Children’s Hospital Medical Center, Cincinnati, OH USA; 24 Division of Neurology, Department of Pediatrics, University of Cincinnati College of Medicine, Cincinnati, OH USA; 25 Rosamund Stone Zander Translational Neuroscience Center, Boston Children’S Hospital, Boston, MA USA; 26 Department of Paediatric Neurology, University Hospitals Leuven, Leuven, Belgium; 27 Department of Development and Regeneration, KU Leuven, Leuven, Belgium; 28 Department of Pediatrics, Koningin Mathilde Moeder- en Kindcentrum, Antwerp University Hospital, Antwerp, Belgium; 29 Department of Translational Neurosciences, University of Antwerp, Antwerp, Belgium; 30 Centre for Medical Genetics, Antwerp University Hospital, Antwerp, Belgium

**Keywords:** Tuberous Sclerosis Complex, TSC-Associated Neuropsychiatric Disorders, TAND, TAND-SQ Checklist, Validity, Reliability

## Abstract

**Background:**

Tuberous Sclerosis Complex (TSC) is a rare multi-system genetic disorder characterised by benign growths in multiple body systems. TSC-Associated Neuropsychiatric Disorders (TAND) are very common in individuals with TSC, but families often struggle to access appropriate clinical care. To address this gap, the new TAND-SQ Checklist allows individuals with TSC or their caregivers to self-report and quantify characteristics of TAND. The 33 items make up seven natural TAND clusters and an eighth cluster reflecting psychosocial difficulties in individuals with TSC and their caregivers. Respondents rate items as having *ever* been present to generate cluster scores (CS), and rate item *severity* (over the last month) on a 10-point scale to generate cluster severity scores (CSS_mean_) and a total TAND severity score (TTSS_mean_). The purpose of this study was to determine the reliability and validity of the CS, CSS_mean_ and TTSS_mean_ of the TAND-SQ.

**Methods:**

A descriptive group design was used. Two convenience samples with existing clinical data were recruited from the TSC Alliance Natural History Database (NHD) in the USA (n = 69), and from the Developmental Synaptopathies Consortium Rare Diseases Clinical Research Network (RDCRN) study based at Boston and Cincinnati Children's Hospitals (n = 23), totalling 92 participants.

**Results:**

Analyses showed good internal consistency for CS (Cronbach’s alphas: 0.67–0.89) and CSS_mean_ (0.76–0.95) with the exception of the eat/sleep cluster. Within the TAND-SQ, most CS and all CSS_mean_ were significantly correlated to corresponding self-reported clinical diagnoses, and the TTSS_mean_ was significantly correlated to a global self-rating of TAND burden (ρ = 0.75; *p* < .001). Significant correlations were observed between the CS and CSS_mean_ and a range of relevant standardised behavioural measures in the RDCRN cohort. The TTSS_mean_ was significantly correlated with global measures of adaptive behaviour (ρ = − 0.75; *p* < .001) and emotional/behavioural difficulties (ρ = 0.71; *p* = .001). All CS were significantly correlated with corresponding diagnoses of autism, ADHD, anxiety disorder, depressive disorder, scholastic difficulties, and neuropsychological difficulties where reported in the RDCRN and NHD cohorts.

**Conclusions:**

Findings provide support for the reliability and validity of the CS, CSS_mean_ and TTSS_mean_ of the TAND-SQ and support their use in clinical decision-making for TAND management and in further research.

**Supplementary Information:**

The online version contains supplementary material available at 10.1186/s13023-025-03642-2.

## Background

Tuberous Sclerosis Complex (TSC) is a rare genetic disorder characterised by benign tumours in various organs of the body, as well as a range of behavioural, psychiatric, intellectual, academic, neuropsychological and psychosocial challenges that have been termed TSC-Associated Neuropsychiatric Disorders, abbreviated as ‘TAND’ [1–3]. TAND are present in most individuals with TSC [3–7] and contribute significantly to the burden of the illness in individuals with TSC and their families [8]. However, there is a substantial and persistent assessment and treatment gap for TAND [3, 6]. To help address this gap, the Neuropsychiatry Panel of the 2012 International TSC Consensus Conference coined the term ‘TAND’ to provide a standard way to refer to these difficulties, and developed a TAND checklist that clinicians could use as a structured memory aid during clinical interviews with families to guide further referral and treatment [1, 3]. The TAND Checklist Lifetime version (TAND-L) was published in 2015 and demonstrated high face validity to researchers, clinicians, and family representatives in the TSC community, good internal consistency in its various sections, and promising evidence of convergent validity with standardised measures of similar constructs [3, 9].

The TAND-L Checklist has been translated into 20 languages and used clinically worldwide [3, 10] . It has also helped to drive research efforts into understanding TAND, leading to significant advancements in the field [11]. Specifically, multiple studies using the TAND-L have revealed that, despite the enormous variability in TAND presentation, TAND behaviours tend to cluster together into natural TAND clusters and profiles of symptom burden [4, 12–14]. Although the TAND cluster groupings have shown some variation across samples, one of the largest natural cluster studies to date described seven natural clusters: autism-like, dysregulated behaviour, eat/sleep, mood/anxiety, neuropsychological, overactive/impulsive, and scholastic [13]. The identification of these clusters has the potential to help clinicians focus their recommendations for further assessment and relevant interventions for TAND, despite the heterogeneity in TAND presentation across individuals with TSC. Also based on this work, international clinical consensus recommendations for the assessment and treatment of TAND were recently formulated and published specifically for each natural TAND cluster, along with core principles and a set of ‘wraparound’ psychosocial cluster recommendations [15].

With the growing use of the TAND-L, members of the TSC community expressed the need for a self-report TAND checklist that they could complete on their own (i.e., not in a clinical interview as with the TAND-L), and that could capture behaviours not only as having ever occurred but also to quantify the severity of TAND difficulties at the time of completion [16]. This new checklist was recently published [17] and called the TAND-SQ Checklist, with ‘S’ referring to self-report, and ‘Q’ to quantified. The TAND-SQ was developed by the TAND consortium, an international consortium of TSC researchers, clinicians, and family members, as part of the TANDem project [16]. Using the TAND-L as a starting point, the team followed an iterative development process resulting in the TAND-SQ, which included four additional behavioural and neuropsychological items in questions 3 and 7, extended and separated sections on psychosocial burden experienced by individuals with TSC (question 8.1) and their caregivers (question 8.2) given the growing evidence of psychosocial burden and stress associated with TSC both in individuals with TSC [18–20] and their caregivers [21–25], a question on helpful strategies (question 12), a question on strengths, skills and talents (question 13), a TAND Cluster Profile (question 14), and quantification using severity ratings for difficulties at the behavioural, scholastic, and neuropsychological levels in questions 3, 6, and 7 of the checklist respectively [17]. The 33 individual TAND items are rated as having *ever* been present to generate cluster scores (CS), and how *severe* the item has been (in the last month) on a 10-point scale to generate cluster severity scores (CSS) and a total TAND severity score (TTSS).

### Validation of the TAND-SQ

The TAND-L and TAND-SQ are ‘checklists’ [26] that have been designed to be used as systematic tools to screen for high frequency TAND difficulties. Importantly, checklists are not psychological tools designed to measure latent clinical conditions or typical screening tools where ‘norms’ or ‘cut-off’ values are calculated to predict a likely disorder (e.g. depressive disorder), or psychological construct (e.g. self-esteem). Instead, *any* item endorsed by families or individuals with TSC may be important for further investigation or intervention, which is the fundamental purpose of a checklist [26, 27]. However, the TAND-SQ *cluster* scores (CS, CSS and TTSS) do represent patterns of functioning and it is unknown how the items within these clusters relate to one another, or how the CS, CSS and TTSS scores relate to measures of similar constructs or relevant clinical diagnoses. Therefore, various validation constructs for these new TAND-SQ scores are relevant and important to consider.

Many different types of validity have been described, depending on the nature of the tool, measurement or type of research. Following a literature review, Flake and colleagues recommended a framework for validation of new measures [28]. The framework specified validation constructs to consider according to three phases of measure development: 1) substantive phase (constructs evaluated during measure development), 2) structural phase (evaluation of constructs internal to the measure), and 3) external phase (evaluation of constructs in the measure against external measures or established criteria). Using this framework as a guide, we identified aspects of validation relevant to the TAND-SQ. These are summarised in Table 1 along with definitions relevant to the TAND-SQ and methods of assessment.

**Table 1:** Key validation constructs used to evaluate the TAND-SQ

Key validation construct	Definition	Method of assessment
**Phase I: substantive phase—constructs evaluated during measure development**		
Face validity	Does the checklist appear to measure the TAND constructs of interest?	Expert review
Content validity	Does the checklist appear to measure *all* the components of relevance to TAND?	Expert review
Response process validity	Is the checklist easy to use, clearly understandable, culturally appropriate to those who will use it?	User review
**Phase II: structural phase—evaluation of constructs internal to the measure**		
Internal consistency	Do the different items on the checklist that propose to measure the same cluster relate to one another?	Cronbach’s alpha ($\alpha$)
Inter-item correlations	Do cluster scores correlate with other specific items or diagnoses also reported on the TAND-SQ?	Spearman correlations (ρ)
**Phase III: external phase—evaluation of constructs in the measure against external measures or established criteria**		
Convergent and discriminant validity	Do checklist scores correlate with other scales meant to capture similar or different behavioural constructs, and relevant clinical or research diagnoses?	Spearman correlations (ρ)

The substantive phase of development of the TAND-SQ was described by Heunis and colleagues [17] where face validity, content validity and response process validity were all assessed as an integral part of the participatory design and development of the TAND-SQ [17]. In that study, a total of 81 participants (23 ‘technical experts’ and 58 ‘lived experts’) provided feedback about comprehensiveness, clarity, ease of use and overall acceptability of the TAND-SQ. Participants gave high ratings for the ‘near-final’ version of the TAND-SQ for all four questions, suggesting good face and content validity as well as response process validity [17]. These findings were similar to those seen in the pilot validation of the TAND-L [9].

The purpose of this study was to perform a detailed examination of other validity constructs relevant to the TAND-SQ described in Phase II and III of Flake and colleagues’ framework [28]. This included an examination of the internal consistency of the items within each cluster, correlations between cluster scores and other items and responses within the TAND-SQ, and convergent and discriminant validity of the CS, CSS, and TTSS of the TAND-SQ in relation to relevant well-known standardised measures and clinical diagnoses outside the TAND-SQ.

## Methods

### Study aim and design

The aim of this study was to validate the scores generated by the new TAND-SQ, in terms of how the items relate to each other (internal consistency), how the scores relate to other items within the TAND-SQ (inter-item correlations), and how they relate to measures and clinical diagnoses in independent datasets (convergent and discriminant validity) using a descriptive group design.

### Study participants

Two convenience samples of participants with existing independent clinical data for comparison purposes were recruited from the TSC Alliance Natural History Database (NHD), and the Developmental Synaptopathies Consortium Rare Diseases Clinical Research Network (RDCRN) study based at Boston Children's Hospital (BCH) and Cincinnati Children's Hospital (CCH) in the USA. The NHD sample reflects a ‘real-world’ clinical dataset, and the RDCRN sample a standardised research dataset. Caregivers of children with TSC under the age of 18, caregivers of adults with TSC who had intellectual developmental levels that prevented them from completing the TAND-SQ themselves, and adults over the age of 18 with TSC who were able to complete the TAND-SQ themselves were all eligible to participate.

*TSC Alliance NHD cohort.* After signing consent, adult individuals with TSC and caregivers of individuals with TSC were invited to complete the TAND-SQ Checklist via the TSC Alliance electronic self-report portal. The TAND-SQ checklist was added to the existing TSC Alliance NHD portal which allows families or individuals to report on their own health outcomes. The NHD also contains clinical data for individuals with TSC captured and entered by TSC Alliance staff members or participating clinics throughout the USA. Of relevance to this study, the NHD includes clinically reported neuropsychiatric diagnostic information, specifically, the presence or absence of autism, attention deficit hyperactivity disorder (ADHD), anxiety or mood disorder, neuropsychological deficits, and school difficulties. The TSC Alliance utilised various recruitment methods to invite participants to the study, including posting information about the project on their website, online community support and Facebook pages, email and in print (e.g., TSC Alliance magazine and flyers at a TSC Alliance sponsored event).

*RDCRN cohort.* Participants from TSC clinics at BCH and CCH were invited to participate if they were enrolled in phase I and/or II of the Developmental Synaptopathies Consortium RDCRN study [29]. From the RDCRN study, participants had detailed evaluation data from a range of standardised and validated assessments, clinical interviews, questionnaires and rating scales that allowed us to compare TAND-SQ scores with valid, standardised assessment tools of relevant behavioural constructs and diagnostic evaluations. Participants were recruited by the primary clinical contacts at the respective sites. The clinicians and their teams who invited the participants all had expertise in working with patients with TSC, were all familiar with the clinical and personal needs of the families and were therefore best positioned to determine whether study participation was appropriate. Families who agreed to participate and signed consent were asked by research co-ordinators at study sites to complete the app-based TAND-SQ Checklist during routine clinical visits using a unique study code.

### Participant characteristics

Participant characteristics are summarised in Table 2. In total, 92 participants took part in the study, 69 from the TSC Alliance NHD cohort and 23 from the RDCRN study cohort. Although the RDCRN study comprised a much larger cohort overall, only participants from two RDCRN sites, BCH and CCH, were invited to participate in the current add-on validation study as part of the TANDem project. Recruitment depended on participants’ availability and consent to participate. Some of the standardised measures used in the RDCRN cohort were age-specific or required a minimum level of language to be valid, so not all measures were completed in all participants. A total of 71 caregivers of child (*n* = 51) and adult (*n* = 20) dependents and 21 individuals with TSC completed a TAND-SQ Checklist. This resulted in 51 TAND-SQ Checklists for children under the age of 18, and 41 checklists for adults 18 years and older. The total sample was almost evenly divided between male and female and levels of intellectual functioning (see Table 2).

**Table 2:** Participant characteristics

Characteristic	Children (< 18 years) *n* = 51	Adults (≥ 18 years) *n* = 41	Total (*n* = 92)
Age			
Mean (SD) (years)	7.63 (4.13)	35.91 (14.90)	20.23 (17.51)
Range (years)	0.66–15.10	18.07–71.93	0.66–71.93
Sex	*n* (% of sample)		
Male	27 (53%)	17 (42%)	44 (48%)
Female	24 (47%)	24 (58%)	48 (52%)
Intellectual ability (as reported in question 5 of the TAND-SQ)	*n* (% of sample)		
Normal/Above average IA	17 (33%)	19 (46%)	36 (39%)
Mild-moderate ID	26 (51%)	11 (27%)	37 (40%)
Severe-profound ID	8 (16%)	11 (27%)	19 (21%)

IA: intellectual ability; ID: intellectual disability

### Ethical review and consent

This study was approved by the Human Research Ethics Committee (HREC) at the University of Cape Town, South Africa (HREC reference number: 849/2020), the site of the principal investigator, and at the Vrije Universiteit Brussel, Belgium (BUN: 1432022000037), the site of the co-principal investigator. It was also approved by the Ethical and Independent Review Services for the Natural History Database Study (protocol number 15039-08) in the United States which permitted use of deidentified clinical data for TSC research at the TSC Alliance, and by the BCH Institutional Review Board IRB (IRB-P00041212). For the BCH and CCH sites, BCH agreed to serve as the reviewing IRB for this study and CCH (IRB number 2022-0421) agreed to cede IRB review to the BCH IRB. All TSC Alliance NHD participants, and BCH and CCH participants were asked to provide informed consent before participating in this study. All participating data collection sites signed a data transfer agreement which allowed for secure sharing of pseudonymised data.

### Behavioural measures and scores

*Measure of TAND: TAND-SQ Checklist.* The TAND-SQ [17] collects information regarding developmental history (question 1), current level of functioning (question 2), behavioural difficulties (question 3), psychiatric disorders (question 4), intellectual ability (question 5), difficulties in learning at school (question 6), neuropsychological difficulties (question 7), and psychosocial challenges for the individual with TSC (question 8.1) and their caregivers (question 8.2). In question 10, respondents are asked to give a summary rating of how much all the difficulties in the checklist have bothered, troubled or distressed the individual and/or family over the last month on a scale from 0 (not at all) to 10 (extremely).

*TAND-SQ scores.* The 33 items in questions 3, 6 and 7 are all associated with one of seven TAND clusters (autism-like, dysregulated behaviour, eat/sleep, mood/anxiety, neuropsychological, overactive/impulsive, scholastic, as outlined in Table 3 [13]. Each item has two parts, a) ‘Has this ever been a problem?’ and b) ‘If yes, how much of a problem has it been over the last month?’. Part (a) requires a Yes/No response, and part (b) requires a severity rating between 0 (not at all) and 10 (extremely). The responses to part (a) and part (b) allow for different scores to be calculated for each TAND cluster. For the purpose of this validation study, we calculated two scores for each cluster - a cluster score (CS), and a mean cluster severity score (CSS_mean_). The CS is calculated as the proportion of items endorsed as ‘Yes’ within a cluster, in response to part (a). The CSS_mean_ is calculated as the mean of all severity ratings for all items within the cluster, in response to part (b). CS and CSS_mean_ scores, as shown below in Eqs. 1 and 2.

1 $$\begin{aligned} Cluster \,Score \left( {CS} \right) =\, & proportion \,of\, items\, endorsed\, as \,Yes\, within\, the\, cluster \\ = & \frac{number\, of\, items\, endorsed\, as\, Yes\, in\, the\, cluster}{{total\, number\, of\, items\, in\, the\, cluster}} \\ \end{aligned}$$

2 $$\begin{aligned} Cluster \,Severity\, Score \left( {CSS_{mean} } \right) =\, & mean\, of\, severity\, ratings\, of\, all\, items\, within \,the\, cluster \\ = & \frac{sum\, of\, severity\, ratings\, for\, all\, items\, within\, the\, cluster }{{total\, number\, of\, items\, in\, the\, cluster}} \\ \end{aligned}$$

**Table 3:** TAND-SQ items linked to TAND clusters and used in the calculation of CS, CSS_mean_ and TTSS_mean_

TAND cluster	TAND-SQ items	Item number
Autism-like (7 items)	Absence or delayed onset of language Repeats words or phrases Poor eye contact Difficulty in peer relationships Repetitive behaviours Rigid or inflexible behaviour Sensory sensitivities*	3h 3i 3j 3k 3l 3m 3n
Dysregulated behaviour (3 items)	Aggressive outbursts Temper tantrums Self-injury	3e 3f 3g
Eat/Sleep (2 items)	Difficulties with eating and/or drinking Difficulties with sleeping	3s 3t
Mood/Anxiety (4 items)	Anxiety Depressed mood Extreme shyness Mood swings	3a 3b 3c 3d
Neuropsychological (10 items)	Motor skills* Language skills* Attention Attention or concentration Dual/multi-tasking Memory Visuo-spatial tasks Executive skills Orientation Processing speed*	7a 7b 7c 3p 7d 7e 7f 7g 7h 7i
Overactive/Impulsive (3 items)	Overactivity/hyperactivity Restlessness or fidgetiness Impulsivity	3o 3q 3r
Scholastic (4 items)	Reading Writing Spelling Mathematics	6a 6b 6c 6d
Psychosocial-Individual (7 items)	Low self-esteem Stress in the family Stress in sibling relationships Parent–child relationship difficulties* Parent-parent/partner relationship difficulties* Connecting with others in the community* Difficulty in career progress*	8.1a 8.1b 8.1c 8.1d 8.1e 8.1f 8.1g
Psychosocial-Caregiver (7 items)	Low self-esteem* Stress in the family* Stress in sibling relationships* Parent–child relationship difficulties* Parent-parent/partner relationship difficulties* Connecting with others in the community* Difficulty in career progress*	8.2a 8.2b 8.2c 8.2d 8.2e 8.2f 8.2g

*Indicates new items in the TAND-SQ not included in the TAND-L or previous cluster analyses

A total TAND severity score (TTSS) can also be calculated as a potential measure of global TAND burden. This is calculated as the *sum* of the severity ratings of all 33 items (TTSS_max_) or as a *mean* of the severity ratings of all 33 items (TTSS_mean_) in questions 3, 6 and 7. For the validation study we used the mean TTSS (TTSS_mean_). The formula is shown in Eq. 3.

3 $$\begin{aligned} Total \,TAND\, Severity\, Score \left( {TTSS_{mean} } \right) = & \,mean\, of\, severity\, ratings\, of\,all\, items\, in \,questions \,3, 6 \,and \,7 \\ = & \frac{sum \,of\,severity \,ratings\, for\, all\, items \,in\, questions \,3, 6 \,and\, 7}{{ total \,number\, of\, items\, in\, questions\, 3, 6 \,and\, 7}} \\ \end{aligned}$$

For all scores calculated (CS out of a total of 1, CSS_mean_ out of a total of 10 and TTSS_mean_ out of a total of 10), higher scores indicate more severe ratings of TAND behaviours.

For the psychosocial clusters for individuals with TSC and their caregivers (questions 8.1 and 8.2 respectively), only a CS could be computed as the items in 8.1 and 8.2 do not include a severity rating. Psychosocial CS were therefore calculated using Eq. 1.

*Independent standardised measures (RDCRN cohort only)*. A range of measures were selected from the RDCRN database deemed relevant to the TAND-SQ clusters based on expert review within the TAND consortium [16]. The selected measures are summarised in Table 4 and included measures of autism features, social communication characteristics, mood, behaviour, intellectual ability and neuropsychological skills, adaptive functioning, and quality of life measured using standard T-scores. Measures of eating and sleeping difficulties were obtained from detailed clinical history information.

**Table 4:** Constructs and standardised measures used for external validation analyses

Construct	Standardised measure scores from RDCRN database*
Autism features and social communication-related characteristics	Autism Diagnostic Observation Schedule-2 (ADOS-2) [30] total score Repetitive Behavior Scale (RBS) [31] total score Short Sensory Profile (SSP) [32] total score Social Responsiveness Scale (SRS) [33] total score and social communication and interaction (SCI) score
Dysregulated behaviour	Child Behavior Checklist (CBCL) [34] externalizing T-score CBCL total T-score Aberrant Behavior Checklist (ABC) [35] irritability T-score
Eating and sleeping difficulties	Diet history questions, report of one or more of the following (Yes/No): non-regular table food, texture modification, high-calorie food, nutritional supplement, special diet, food aversion and food allergies Sleep history question: report of disrupted sleep patterns (Yes/No)
Mood and anxiety symptoms	CBCL internalizing T-score CBCL total T-score ABC lethargy/social withdrawn T-score
Neuropsychological skills	Stanford-Binet-5 [36] abbreviated IQ score Wechsler processing speed [37] composite score Peabody Picture Vocabulary Test (PPVT) total score [38] Expressive Vocabulary Test (EVT) total score [39] Beery-Buktenica test of Visual-Motor Integration (VMI) [40] total score Vineland Adaptive Behavior Scale-3 (VABS-3) [41] Adaptive behavior composite (VABS-3 ABC) standard score
Overactive/Impulsive behaviour	CBCL externalizing T-score CBCL total T-score ABC hyperactivity T-score ABC irritability T-score
Scholastic skills	Stanford-Binet-5 abbreviated IQ score VABS-3 ABC standard score
Psychosocial burden	Child/individual & family quality of life measure (CFQL- 2 or IFQL) [42] total score
Overall behavioural functioning	CBCL total score VABS-3 ABC standard score

*For most measures, higher scores indicate greater severity of behavioural difficulties, except for the measures of neuropsychological skills, scholastic skills, psychosocial burden, and the Short Sensory Profile (SSP) in the autism category, where higher scores indicate better functioning.

*Independent reported clinical diagnoses (NHD and RDCRN cohorts).* Clinical diagnoses of autism, ADHD, anxiety and depressive disorder were reported as present or absent for both the RDCRN and NHD cohorts. The diagnoses reported for the NHD cohort were recorded based on clinical assessments and recorded by trained TSC Alliance staff members or participating TSC clinic research team members. The presence or absence of school difficulties, and neuropsychological deficits were also reported for the NHD cohort. The diagnoses in the RDCRN cohort were based on the DSM-5 Checklist for depressive disorder, anxiety, and ADHD. The RDCRN autism diagnosis used in this study was an expert team consensus diagnosis using DSM-5 criteria supplemented by a collection of measures including the Autism Diagnostic Interview-Revised (ADI-R), Autism Diagnostic Observation Schedule-2 (ADOS-2) and an Autism Clinical Certainty score.

### Data collection procedures

*TSC Alliance NHD cohort.* Following provision of informed consent, participants from the TSC Alliance completed the TAND-SQ Checklist through a secure electronic portal on the Studytrax platform. TAND-SQ responses collected through the TSC Alliance portal were linked to existing clinical data stored in the NHD. Pseudonymised TAND-SQ data and selected NHD data were then shared with the research team via a secure electronic portal in accordance with a formal data sharing agreement between all participating sites and the TAND consortium.

*RDCRN cohort.* BCH and CCH participants used the TAND Toolkit App, developed as part of the TANDem project [16], to collect data. Following consent to participate in the study, app users had to download the app, register using a unique study code, provide in-app informed consent, accept the Privacy Policy and Terms of Use, and were then able to complete a TSC Story, a TAND-SQ Checklist and an App Feedback Form. Pseudonymised app data were then linked via the unique study code to pseudonymised detailed phenotyping and neuropsychological testing data collected at BCH and CCH as part of the RDCRN study. Data were shared with the research team via a secure electronic portal for analysis in accordance with a formal data-sharing agreement between all participating sites and the TAND consortium.

### Data analysis

All data were imported from Excel to IBM SPSS version 29.0.0.0 for the following analyses:

*Internal consistency of TAND-SQ CS and CSS*_*mean*_ Cronbach’s alpha ($\alpha$) was used to determine internal consistency of the items within the CS and CSS_mean_ of the seven previously identified natural TAND-SQ clusters [13], and the CS only for the new wraparound psychosocial cluster for the individuals with TSC and for caregivers [15]. Given that new items were added to the TAND-SQ in the autism-like (one new item), neuropsychological (three new items), psychosocial-individual (four new items), and psychosocial-caregiver (seven new items) clusters [17], Cronbach’s alphas ($\alpha$) were calculated and compared for the CS using only the original items from the TAND-L used in the previous cluster analysis studies, and for the CS using all items in the TAND-SQ including all new items. These results are presented in the Supplemental Material (see Supplemental Table 1).

*Correlations of TAND-SQ CS, CSS*_*mean*_* and TTSS*_*mean*_* with other items within the TAND-SQ.* Inter-item correlations between the TAND-SQ CS, CSS_mean_ and TTSS_mean_ relative to other items within the TAND-SQ were examined in two ways, 1) we used Spearman point-biserial correlations to determine whether the TAND-SQ CS and CSS_mean_ were related to relevant self-reported clinical diagnoses reported in question 4 of the TAND-SQ, and 2) we examined the relationship between the TTSS_mean_ and the global rating of how much TAND difficulties had troubled the individual and/or family assessed in question 10 using a Spearman’s correlation ($\rho$).

*Convergent validity of TAND-SQ CS, CSS*_*mean*_* and TTSS*_*mean*_ 1) We used Spearman’s correlations ($\rho$) to determine the relationship between the TAND-SQ CS and CSS_mean_ and a range of relevant standardised measures within the RDCRN cohort as described in Table 4. We examined the relationship between the TTSS_mean_ and two global measures of functioning, the VABS-3 ABC and the CBCL Total T-score using Spearman’s correlations ($\rho$). 2) The relationship between the TAND-SQ CS and CSS_mean_ and clinical diagnoses reported in the TSC Alliance NHD and RDCRN cohorts were examined using Spearman’s rho point-biserial correlations.

*Convergent validity of TAND-SQ clinical diagnoses relative to externally reported clinical diagnoses.* We also examined the relationships between clinical diagnoses reported in the TAND-SQ in question 4, and those reported in the TSC Alliance NHD (*n* = 69) and RDCRN (*n* = 23) cohorts using chi-squared (χ^2^) calculations for categorical data.

## Results

### Internal consistency of TAND-SQ CS and CSS_mean_

Cronbach’s alpha ($\alpha$) for TAND-SQ CS and CSS_mean_ using all items included in the TAND-SQ are presented in Table 5. Results showed that, except for the eat/sleep cluster, all CS had internal consistency ≥ 0.67 and all CSS_mean_ had an internal consistency ≥ 0.76. Given this observation, the eat/sleep cluster was not used in any further analyses. For all clusters, internal Cronbach’s alphas ($\alpha$) were larger for CSS_mean_ responses than the CS responses. Please refer to the Supplemental Material for our rationale for including all items in these calculations, and comparison of results presented in Supplemental Table 1.

**Table 5:** Internal consistency of TAND-SQ cluster scores (CS) and cluster severity scores (CSS_mean_) (*n* = 92)

TAND Cluster	Cronbach’s alpha ($\alpha$) *	
	Cluster scores (CS)	Cluster severity scores (CSS_mean_)
Autism-like (7 items)	.82	.84
Dysregulated behaviour (3 items)	.67	.89
Eat/Sleep (2 items)	.37	.57
Mood/Anxiety (4 items)	.67	.80
Neuropsychological (10 items)	.89	.94
Overactive/Impulsive (3 items)	.67	.76
Scholastic (4 items)	.81	.95
Psychosocial–Individual (7 items) (*n* = 92)	.78	–
Psychosocial–Caregiver (7 items) (*n* = 51)	.82	–

*$\alpha$ values of 0.70 and above are considered acceptable

### Correlations of TAND-SQ CS, CSS_mean_ and TTSS_mean_ with other items within the TAND-SQ

Spearman’s point-biserial correlations between the TAND-SQ scores and relevant corresponding clinical diagnoses reported in question 4 of the TAND-SQ are presented in Table 6. All but one of the CS and all the CSS_mean_ were significantly correlated with the self-report of a relevant clinical diagnosis (i.e., autism, ADHD, anxiety disorder, depressive disorder). The psychosocial CS for individuals with TSC were also significantly associated with the diagnosis of both anxiety and depressive disorder with small effects. Please refer to the Supplemental Material for an expanded version of this table and discussion of discriminant validity (Supplemental Table 2).

**Table 6:** Spearman correlations ($\rho$) between TAND-SQ CS, CSS_mean_ and clinical diagnoses as reported in the TAND-SQ for the total group (*n* = 92)

TAND cluster	Diagnosis reported in question 4 of TAND-SQ	Correlation with cluster score (CS) ρ (*p* value)	Correlation with cluster severity score (CSS_mean_) ρ (*p* value)
Autism-like	Autism	**0.75 (*p* < .001)**	**0.75 (*p* < .001)**
OveractiveIimpulsive	ADHD	0.09 (*p* = .405)	**0.29 (*p* = .007)**
Mood/Anxiety	Anxiety disorder	**0.41 (*p* < .001)**	**0.54 (*p* < .001)**
Mood/Anxiety	Depressive disorder	**0.29 (*p* = .005)**	**0.33 (*p* = .001)**
Psychosocial–Individual	Anxiety disorder	**0.24 (*p* = .023)**	**–**
Psychosocial–Individual	Depressive disorder	**0.26 (*p* = .013)**	**–**

ADHD: attention deficit hyperactivity disorder; values of *p* < .05 are considered significant; significant correlations are presented in bold

The TTSS_mean_ was significantly associated with participants’ global self-rating of TAND impact in question 10 with a medium-large effect size: Spearman’s $\rho$ = 0.75 (p < 0.001).

### Convergent validity of TAND-SQ CS, CSS_mean_ and TTSS_mean_

*Convergent validity with* s*tandardised behavioural measures.* The relationships observed between the TAND-SQ CS, CSS_mean_ and selected conceptually relevant standardised behavioural measures available for the RDCRN cohort (*n* = 23) are summarised in Table 7. All Spearman rho correlations ($\rho$) were in the expected direction (i.e., positive correlations were observed where higher scores in both measures reflect worse functioning, and negative correlations where higher scores on one measure reflected better functioning). The autism-like CS and CSS_mean_ were both significantly correlated with a range of standardised measures designed to identify characteristics associated with autism, including autism characteristics (ADOS-2 total score) [30], social communication differences (Social Responsiveness Scale Total Score) [33], and sensory behaviours (Short Sensory Profile) [32]. The autism-like CS (but not the CSS_mean_) was significantly correlated with a measure of repetitive behaviour (Repetitive Behavior Scale) [31].

**Table 7:** Spearman’s rho correlations (ρ) between TAND-SQ cluster scores (CS), mean cluster severity scores (CSS_mean_) and external standardised measures

RDCRN standardised measures	Spearman’s rho ρ (*p* value*)	
	TAND-SQ scores	
	Cluster Score (CS)	Cluster Severity Score (CSS_mean_)
	**Autism-like cluster**	
ADOS-2 total score (*n* = 15)	**0.55 (*p* = .033)**	**0.58 (*p* = .024)**
RBS total score (*n* = 15)	**0.55 (*p* = .034)**	0.39 (*p* = .155)
SSP total score (*n* = 22)	**− 0.59 (*p* = .004)**	**− 0.53 (*p* = .011)**
SRS total score (*n* = 21)	**0.57 (*p* = .007)**	**0.56 (*p* = .009)**
SRS SCI score (*n* = 18)	**0.62 (*p* = .006)**	**0.66 (*p* = .003)**
	**Dysregulated behaviour cluster**	
CBCL externalizing T-score(*n* = 18)	**0.61 (*p* = .007)**	0.46 (*p* = .053)
CBCL total T-score (*n* = 18)	**0.54 (*p* = .020)**	**0.69 (*p* = .002)**
ABC irritability T-score(*n* = 19)	**0.75 (*p* < .001)**	**0.67 (*p* = .002)**
	**Mood/Anxiety cluster**	
CBCL internalizing T-score (*n* = 18)	**0.56 (*p* = .020)**	**0.64 (*p* = .006)**
CBCL total T-score (*n* = 18)	**0.71 (*p* = .001)**	**0.61 (*p* = .008)**
ABC lethargy/withdrawn T-score (*n* = 19)	0.45 (*p* = .054)	0.41 (*p* = .084)
	**Neuropsychological cluster**	
SB-5 abbreviated IQ score (*n* = 16)	**− 0.76 (*p*< .001)**	**− 0.78 (*p* < .001)**
Wechsler PSI composite score (* n* = 10)	**− 0.73 (*p* = .018)**	**− 0.77 (*p* = .010)**
PPVT total score (*n* = 14)	**− 0.77 (*p* = .001)**	**− 0.83 (*p* < .001)**
EVT total score (*n* = 16)	**− 0.71 (*p* = .002)**	**− 0.80 (*p* < .001)**
VMI total score (*n* = 18)	− 0.42 (*p* = .083)	**− **0.41 (*p* = .094)
VABS-3 ABC standard score (*n* = 22)	**− 0.83 (*p* < .001)**	**− 0.80 (*p* < .001)**
	**Overactive/Impulsive cluster**	
CBCL externalizing T-score (*n* = 18)	**0.67 (*p* = .002)**	**0.63 (*p* = .005)**
CBCL total T-score (*n* = 18)	**0.58 (*p* = .012)**	**0.69 (*p* = .001)**
ABC hyperactivity T-score (*n* = 19)	**0.67 (*p* = .002)**	**0.64 (*p* = .003)**
ABC irritability T-score (*N* = 19)	**0.77 (*p* < .001)**	**0.84 (*p* < .001)**
	**Scholastic cluster**	
SB-5 abbreviated IQ score (*n* = 16)	**− 0.81 (*p* < .001)**	**− 0.80 (*p* < .001)**
VABS-3 ABC standard score (*n* = 22)	**− 0.60 (*p* = .003)**	**− 0.65 (*p* = .001)**
	**Psychosocial cluster**	
CFQL Total score		
Individuals (*n* = 17)	**− 0.63 (*p* = .006)**	–
Caregivers (*n* = 12)	− 0.42 (*p* = .170)	–
	**Total TAND severity score (TTSS**_**mean**_**)**	
CBCL total score (*n* = 18)	**0.71 (*p* = .001)**	
VABS-3 ABC standard score (*n* = 22)	**− 0.75 (*p* < .001)**	

*ADOS-2* autism diagnostic observation schedule-2; *RBS* repetitive behavior scale; *SSP* short sensory profile; *SRS* social responsiveness scale; *SCI* social communication and interaction; *CBCL* child behavior checklist; *ABC* aberrant behavior checklist; *SB* stanford-binet-5; *PPVT* peabody picture vocabulary test; *EVT* expressive vocabulary test; *VMI* Beery-Buktenica tests of visual-motor integration-6; *VABS-3 ABC* Vineland-3 adaptive behavior composite; *PSI* processing speed index

*Values of *p* < .05 are considered significant; significant correlations are presented in bold; negative correlations were observed for measures where higher scores reflect better functioning.

The dysregulated behaviour CS and CSS_mean_ were significantly correlated with the CBCL Total Score [34] and the ABC Irritability subscale [35], while only the CS was correlated with the CBCL Externalising T-score. The mood/anxiety CS and CSS_mean_ were associated with the CBCL Internalizing T-score and CBCL total score but not the ABC Lethargy/Withdrawn subscale. The neuropsychological CS and CSS_mean_ were significantly correlated with intellectual ability (Stanford-Binet-5 Abbreviated IQ Score) [36], processing speed (Wechsler Processing Speed Index Composite Score) [37], receptive vocabulary (PPVT) [38], expressive vocabulary (EVT) [39], and overall adaptive functioning (VABS-3 ABC Score) [41], but not with the Beery-Buktenica Test of Visual Motor Integration [40]. The overactive/impulsive CS and CSS_mean_ were significantly associated with the CBCL Externalizing T-score, CBCL Total Score, and the ABC Hyperactivity and Irritability Scores. Similar to the neuropsychological cluster, the scholastic CS and CSS_mean_ were significantly associated with intellectual ability (Stanford-Binet-5 Abbreviated IQ Score) and overall adaptive functioning on the VABS-3 ABC Score.

The two new psychosocial CS for the individuals with TSC (as rated by themselves or their caregivers; question 8.1) and for caregivers (question 8.2), were examined in relation to scores on the Child and Family Quality of Life measure, second edition (CFQL-2) [42], available for 17 individuals with TSC and 12 caregivers. The findings showed that the psychosocial burden reported for individuals with TSC (question 8.1 of the TAND-SQ) was significantly negatively correlated with the CFQL-2 Total Score. This relationship was not significant for the caregiver psychosocial CS in this small sample. Finally, the TTSS_mean_ showed significant correlations in the predicted direction with two global measures of functioning, one of adaptive behaviour (VABS-3 ABC) and the other of emotional and behavioural difficulties (CBCL Total Score), both with medium to large effect sizes.

*Convergent validity with clinical diagnoses.* Spearman correlations ($\rho$) examining the relationship between TAND-SQ CS, CSS_mean_ and clinical diagnoses reported in the TSC Alliance NHD and RDCRN cohorts are presented in Table 8. Results show significant associations between all TAND-SQ CS and independently reported clinical diagnoses in the NHD and RDCRN cohorts. Three of the six CSS_mean_ were associated with clinical diagnoses reported on the NHD. Only the autism-like CS and CSS_mean_ could be examined in the RDCRN cohort as autism was the only clinical diagnosis reported in this cohort.

**Table 8:** Spearman biserial correlations (ρ) between TAND-SQ Cluster Scores (CS), mean Cluster Severity Scores (CSS_mean_) and externally reported clinical diagnosis in the TSC Alliance NHD and RDCRN cohorts

TAND cluster	Independent diagnosis	Spearman’s ρ for CS (*p* value)	Spearman’s ρ for CSS_mean_ (*p*-value)
**NHD Cohort**			
Autism-like	Autism (*n* = 50)	**0.64 (*p* < .001)**	**0.55 (*p* < .001)**
Mood/Anxiety	Anxiety disorder (*n* = 48)	**0.34 (*p* = .017)**	0.26 (*p* = .079)
Mood/Anxiety	Depressive disorder (*n* = 48)	**0.42 (*p* = .003)**	0.23 (*p* = .113)
Neuropsychological	Neuropsychological deficit (*n* = 21)	**0.50 (*p* = .022)**	**0.43 (*p* = .049)**
Overactive/Impulsive	ADHD (*n* = 48)	**0.34 (*p* = .017)**	0.23 (*p* = .113)
Scholastic	School difficulties (*n* = 48)	**0.40 (*p* = .011)**	**0.37 (*p* = .011)**
Psychosocial–Individual	Anxiety disorder (n = 48)	**0.31 (*p* = .035)**	**–**
Psychosocial–Individual	Depressive disorder (n = 48)	**0.39 (*p* = .006)**	**–**
**RDCRN Cohort**			
Autism-like	Autism (*n* = 23)	**0.48 (*p* = .019)**	0.40 (*p* = .058)

NHD: natural history database; ADHD: attention deficit hyperactivity disorder; RDCRN: rare diseases clinical research network; values of *p* < .05 are considered significant; significant correlations are presented in bold

### Convergent validity of the TAND-SQ clinical diagnoses relative to externally reported clinical diagnoses

The relationship between clinical diagnoses reported in question 4 of the TAND-SQ and those reported in the TSC Alliance NHD and RDCRN cohorts are presented in Table 9. Chi-squared statistics revealed that clinical diagnoses of autism, depressive disorder and anxiety disorder recorded in the TAND-SQ and in the NHD and RDCRN cohorts were significantly associated. It is noteworthy that except for the autism diagnosis in the RDCRN cohort based on a systematic research protocol, there were many more clinical diagnoses reported on the TAND-SQ than on the NHD or the RDCRN.

**Table 9:** Relationship between diagnostic reporting on the TAND-SQ (question 4) and the TSC Alliance NHD and RDCRN cohorts using chi-squared analysis

Clinical diagnosis	Number of diagnoses by cohort	Number of diagnoses reported in TAND-SQ (question 4)	Chi-squared statistic	*p* value
**TSC Alliance NHD (n = 69)**				
Autism	Yes = 19	Yes = 30	**34.10**	** < .001**
ADHD	Yes = 11	Yes = 17	7.12	.068
Anxiety disorder	Yes = 10	Yes = 23	**9.78**	**.021**
Depressive disorder	Yes = 4	Yes = 13	**23.03**	** < .001**
**RDCRN (n = 23)**				
Autism	Yes = 12	Yes = 13	**12.61**	** < .001**
ADHD	Yes = 0	Yes = 4	**–**	
Anxiety disorder	Yes = 0	Yes = 5	**–**	
Depressive disorder	Yes = 0	Yes = 1	**–**	

ADHD: attention deficit hyperactivity disorder; values of *p* < .05 are considered significant and are presented in bold

## Discussion

In this study we examined the internal consistency and convergent validity of the new TAND-SQ Checklist to complement the previously published data on face, content, and response process validity [17]. We focused on investigating validity for three TAND-SQ scores, the CS, CSS_mean_ and TTSS_mean_ relative to other questions on the TAND-SQ itself and to independently reported performance on widely-used standardised rating scales and clinical diagnostic data in two participant cohorts. We also examined validity of the clinical diagnoses reported in question 4 of the TAND-SQ against clinical diagnoses reported in the RDCRN and TSC Alliance NHD cohorts.

With respect to internal consistency, the findings showed that, with the exception of the eat/sleep cluster, the TAND-SQ items making up the TAND clusters showed acceptable internal consistency, both for the CS and CSS_mean_ scores. This lends support to the addition of the new items to the TAND-SQ in questions 3, 7, 8.1 and 8.2, that were not in the TAND-L or in the original TAND cluster analyses [17]. The low internal consistency within the eat/sleep cluster was not unexpected. Although the two individual items had been linked with each other in the eat/sleep cluster [13], the two items have also been linked closely with other clusters, for example, sleep difficulties with the mood/anxiety cluster and eating difficulties with the autism-like cluster [12, 14]. Sleep difficulties in particular may be driven by biological factors related to the mTOR pathway [43], as well as to the presence of epilepsy and use of epilepsy medications [44].

With respect to validity of the TAND-SQ scores relative to other items within the TAND-SQ, most of the TAND-SQ CS and CSS_mean_ were associated with corresponding relevant self-reported clinical diagnoses reported in question 4 of the TAND-SQ (apart from the overactive/impulsive CS and a reported diagnosis of ADHD). Also, we found that the TTSS_mean_ related significantly to a self-reported overall TAND burden score in question 10 of the TAND-SQ. These findings suggest promising validity of the new CS, CSS_mean_ and TTSS_mean_ scores within the TAND-SQ, and that participants’ responses were consistent in reporting TAND symptom burden throughout the different sections of the TAND-SQ. These findings provide support for the new scores on the TAND-SQ, and the TAND-SQ as a valid tool for screening for TAND.

With respect to convergent validity of the TAND-SQ scores against measures external to the checklist, findings showed that TAND-SQ CS and CSS_mean_ were both significantly associated with a range of relevant behavioural constructs as measured on standardised assessment measures available in the RDCRN cohort. Of note, the autism-like cluster CS and CSS_mean_ were significantly associated with measures of social communication skills, sensory sensitivities, and a composite measure of autism characteristics (ADOS-2). The CS and CSS_mean_ of the dysregulated behaviour cluster, mood/anxiety cluster, and overactive/impulsive clusters were all closely associated with the CBCL Total T-score and the Irritability subscale of the ABC. The neuropsychological and scholastic cluster CS and CSS_mean_ were both closely related to measures of intellectual ability (Stanford-Binet-5) and adaptive behaviour (VABS-3 ABC). The psychosocial CS for individuals with TSC was also significantly associated with a standardised measure of quality of life (CFQL-2). Finally, the TTSS_mean_ was significantly associated with two global indices of behaviour, specifically adaptive behaviour (VABS-3 ABC) and emotional and behavioural difficulties (CBCL Total Score), in the RDCRN cohort. These findings suggest that the TAND-SQ CS, CSS_mean_ and TTSS_mean_ do represent valid behavioural constructs and provide support for the TAND clusters originally identified via data-driven cluster analytic methods [4, 13, 14].

With regards to the association between the TAND-SQ and externally reported clinical diagnoses, all TAND-SQ CS (with the exception of the eat/sleep cluster) showed robust relationships with clinical diagnoses reported in a ‘real-world’ clinical dataset (TSC Alliance NHD) and systematic research dataset (RDCRN), while the CSS_mean_ showed a more mixed picture. As many TAND-SQ items may map onto a given psychiatric diagnosis, it is not entirely unexpected that the TAND-SQ CSS_mean_ may not map as neatly onto externally reported psychiatric diagnoses as they do on individual standardised measures of functioning. The mood/anxiety cluster contains items relevant to both depressive disorder and anxiety disorder, which may have influenced those correlations. In addition, a diagnosis and the CS both capture lifetime presence of symptoms whereas the CSS measures severity of symptoms just within the last month. This severity rating could be impacted by numerous factors including successful treatment, for example, of depression or anxiety.

Taken together, these findings showed that the self-reported TAND-SQ CS and CSS_mean_ captured important indices of behavioural functioning similar to those assessed by standardised measures that include both proxy or self-report questionnaires (for example the CBCL), as well as direct observation (for example, the ADOS-2) and clinical expertise (for example, the systematic research diagnosis of autism). These positive findings regarding convergent validity are similar to those of Müller and colleagues [45], in their validation of the TSC-specific Patient-Reported Outcome Measure (TSC-PROM). The TSC-PROM is a recently published measure for adults that captures the impact of TSC on physical functions, mental functions, activity and participation, and the social support individuals with TSC receive. They found good evidence of convergent validity for the Mental Functions domain of the TSC-PROM in the form of moderate to strong correlations with the CBCL in both the self and proxy versions of the TSC-PROM. Notably, many of the items in the Mental Functions domain overlap with items in the TAND-SQ [45].

### Clinical implications of findings

Our findings suggest that the CS and CSS_mean_ of the TAND-SQ have sufficient validity to guide clinical decision-making. Specifically, they could be used to inform decisions for referrals for formal evaluations of areas of difficulty reflected by the TAND clusters, for example autism-like behaviour, or overactive/impulsive behaviour, where appropriate management options can be considered for the family or individual with TSC. This is in line with the recently published TAND-cluster focused clinical consensus recommendations for TAND [15] and we are encouraged by these findings that support the TAND-SQ as a helpful tool for promoting implementation of these recommendations. In addition, in the event of high TAND burden profiles, which is likely in up to 39% of individuals with TSC [4], we believe the TAND CSS_mean_ generated by the TAND-SQ will be very valuable for families and clinicians to *prioritise* their next step evaluations based on which clusters are presenting the greatest burden at the time of completion. On the other hand, for individuals with low burden profiles, or indeed for example only one TAND behaviour, such as sleep difficulties, that one item should still be followed up with appropriate referrals for evaluation and management.

The TAND-SQ is a new tool that has been deliberately developed for flexible use by families. This may include monthly monitoring of specific symptoms or clusters of concern, in preparation for regular clinic visits, and / or at a minimum, as part of the recommended annual review of TAND [15, 46]. We also suggest that if clinicians have to date asked families to complete the TAND-L independently (i.e., not as a clinical interview), we recommend that they ask families to complete the TAND-SQ (instead of the TAND-L) before each clinical visit. Families can then share their results with the clinician to guide their discussion. We strongly recommend that this should occur at the very least on an annual basis as outlined in the updated international TSC surveillance and management recommendations [46] and in the international consensus recommendations for identification and treatment of TAND [15]. We recommend that clinicians also encourage families to complete the TAND-SQ more regularly if, for example, there is a sudden change in TAND symptoms, to monitor changes when a new medication or behavioural treatment has been initiated, or for monthly monitoring of symptoms or clusters of particular concern. In a manner similar to the TAND-L, we have started a process of providing training to clinical teams and the broader TSC community on use of the TAND-SQ. As families begin to access the TAND-SQ through the TAND Toolkit App [16] we anticipate that the TSC community is likely to play an important role in educating their clinical teams about TAND when they share completed TAND-SQ Checklists with them.

### Implications for future research

From a research perspective, the new quantification scale included in the TAND-SQ allowed for the calculation of TAND *severity* for the first time for clusters (CSS_mean_) and all TAND (TTSS_mean_). This was in response to stated needs within the TSC community to have a sense of how severe their TAND difficulties are impacting them at any one time. We are therefore encouraged by the internal consistency and convergent validity of these new TAND CSS_mean_ and TTSS_mean_ scores. The results support the need for further research to explore the stability of severity ratings of individual items, cluster severity scores (CSS_mean_) and mean TAND severity scores (TTSS_mean_) over time. This would allow for the examination of whether individual item severity, CSS_mean_ and TTSS_mean_ can reliably track changes in TAND over time, and to determine how to define clinically meaningful changes that could support use of the TAND-SQ as an outcome measure in clinical practice and research. If successful, the TAND-SQ would make a valuable contribution as an outcome measure for TAND interventions. The TAND Cluster Profile (question 14) of the recently published TAND-SQ [17] currently generates only the CS for each cluster, and these CS exclude the new items in the autism-like, neuropsychological and psychosocial clusters (in questions 3, 7 and 8, see Table 3). The findings of this study suggest that the TAND Cluster Profile could be expanded to include all new items in future revisions of the TAND-SQ. Further research is also needed to consider how the CSS_mean_ can be used to inform the TAND Cluster Profile and help prioritise and guide next steps in care.

Another novel aspect of the TAND-SQ is the two new separate psychosocial CS now available for individuals with TSC (question 8.1) and their caregivers (question 8.2). As the growing research base has highlighted the psychosocial burden of TSC and TAND on individuals [18–20] and their caregivers [21–25], the TAND consortium recognised a need to capture psychosocial burden in the TAND-SQ in a more comprehensive way for both individuals with TSC and their caregivers separately. This was a notable extension of the TAND-SQ relative to the TAND-L [17]. The findings in this study suggest that the two psychosocial CS have good internal consistency (Cronbach alpha, $\alpha$). The psychosocial CS for individuals also showed small but significant correlations with a self-reported diagnosis of anxiety disorder and depressive disorder. The psychosocial CS for individuals with TSC showed promising convergent validity in its significant relationship with the Child and Family Quality of Life Scale (CFQL-2), as well as significant correlations with independent clinical diagnoses of anxiety disorder and depressive disorder. This is a promising start to supporting use of the psychosocial CS to identify psychosocial burden for individuals and families. More research is needed to understand these new psychosocial CS as measures of psychosocial burden and to examine their relationships with other TAND symptoms and caregiver characteristics. Psychosocial burden was not included in the original cluster research [13, 14] and therefore the items in this cluster were not quantified like the other behavioural cluster items in the TAND-SQ. As the psychosocial CS are showing evidence of convergent validity, future revisions of the TAND-SQ may include adding a severity quantification to these items in question 8.1 and 8.2 of the TAND-SQ. Most importantly, as psychosocial difficulties in individuals with TSC and their caregivers become better understood, there is a need for future research to develop and evaluate interventions to support improved psychosocial functioning of individuals with TSC and their caregivers.

To empower families through the use of technology, the TAND-SQ has been embedded into a smartphone app (TAND Toolkit App) along with a TAND Toolkit containing evidence-informed recommendations for intervention for the TSC community [16]. Our findings regarding the feasibility and acceptability of the TAND Toolkit App are currently being prepared for publication. The TAND-SQ is also being used in new research to track TAND over time in order to determine TAND trajectories and their predictors.

### Study limitations

This study has limitations that must inform interpretation of the results. The ultimate size of the sample was smaller than initially planned, particularly for the RDCRN cohort, resulting in reduced power to detect small or medium effect sizes for many of the analyses in this cohort. Thus, only medium-large effect sizes are likely to have been detected and results need to be replicated in larger samples. Also, many analyses were conducted on a relatively small dataset thus raising the risk of type 1 error (false positive findings). However, we attempted to mitigate this risk and the impact of possible outliers in the small sample size by using only non-parametric analysis methods (Spearman’s correlations). A second noteworthy limitation is the time frame between data collection in the TSC Alliance NHD and RDCRN datasets and completion of the TAND-SQ for this study. We propose that this may explain, for example, the many more psychiatric diagnoses reported on the TAND-SQ than in either the TSC Alliance NHD or the RDCRN datasets. While we were pleased that most of the psychiatric diagnoses reported in the TAND-SQ were significantly associated with those reported in the NHD and RDCRN, it would be beneficial for results to be replicated with concurrent independent diagnostic assessments to confirm these relationships.

We are aware of potential barriers to the implementation of the TAND-SQ across diverse healthcare settings, including cultural and linguistic considerations. For this reason, we have intentionally partnered with the TSC community from multiple countries in the development of the TAND-SQ to ensure a product that will be of use and interest to them. The embedding of the TAND-SQ into a smartphone app for families is also a step to promote its use within the global TSC community. Finally, from our experience translating the TAND-L Checklist, we have a system in place to manage community-led TAND-SQ translations. We believe the global TSC community will play an important role in driving the translation and possible adaptation of the TAND-SQ for global use.

## Conclusion

The TAND-SQ was developed in response to stated needs by the TSC community for a TAND Checklist that could be completed by individuals with TSC and their families themselves, and that could also quantify TAND difficulties. The TAND-SQ thus contains a new quantification rating for items related to natural TAND clusters, and separate expanded psychosocial questions for individuals with TSC and their caregivers. Responses on the TAND-SQ can now be used to calculate a range of TAND scores, specifically CS, CSS_mean_ and TTSS_mean_ for the previously identified natural TAND clusters and separate CS for psychosocial burden for individuals and caregivers. This study found good internal consistency for the CS and CSS_mean_ and evidence of convergent validity for the CS, CSS_mean_ and TTSS_mean_ with other questions within the TAND-SQ, as well as with other standardised behavioural measures and clinical diagnoses independent of the TAND-SQ across two different cohorts. These findings support the use of the TAND-SQ scores by clinicians and families to guide and prioritise clinical decision-making regarding further evaluations and treatment for TAND. The findings also add to previous demonstrations of good face validity, content validity, and response process validity according to expert and user review in the initial development phase of the checklist [17]. Findings also point towards the need for further research to examine the TAND-SQ as a potential measure of TAND severity over time and in response to TAND interventions.

## Supplementary Information

Additional file 1.

## Data Availability

The datasets used and/or analysed during the current study are available from the corresponding author on reasonable request.

## Abbreviations

ABC: Aberrant Behavior Checklist
ADHD: Attention deficit hyperactivity disorder
ADI-R: Autism Diagnostic Interview-Revised
ADOS-2: Autism Diagnostic Observation Schedule-2
BCH: Boston Children’s Hospital
BUN: Vrije Universiteit Brussel Ethics Committee
CBCL: Child Behavior Checklist
CCH: Cincinnati Children’s Hospital
CFQL-2: Child/Individual and Family Quality of Life measure
CS: Cluster Score(s)
CSS_mean_: Cluster Severity Score(s)
DCDQ: Developmental Coordination Disorder Questionnaire
DSM-5: Diagnostic and Statistical Manual-5
EVT: Expressive Vocabulary Test
HREC: Human Research Ethics Committee, University of Cape Town
IA: Intellectual ability
IBM SPSS: IBM Statistical Package for the Social Sciences
ID: Intellectual disability
IRB: Independent Review Board
NHD: Natural History Database of the TSC Alliance, US
PPVT: Peabody Picture Vocabulary Test
PSI: Processing Speed Index
RBS: Repetitive Behavior Scale
RDCRN: Rare Diseases Clinical Research Network
SB-5: Stanford Binet-5
SCI: Social Communication and Interaction
SRS: Social Responsiveness Scale
SSP: Short Sensory Profile
TAND: TSC-Associated Neuropsychiatric Disorders
TAND-L Checklist: TAND-Lifetime Checklist
TAND-SQ Checklist: Self-report Quantified Checklist for TAND
TSC: Tuberous Sclerosis Complex
TSC-PROM: Tuberous Sclerosis Complex-Patient Reported Outcome Measure
TTSS_mean_: Total TAND Severity Score
VABS-3: Vineland Adaptive Behavior Scale-3
VABS-3 ABC: Adaptive Behavior Composite of the VABS-3
VMI: Beery-Buktenica Test of Visual-Motor Integration
