# Supplementary material for: Validation of the self-report quantified Tuberous Sclerosis Complex-Associated Neuropsychiatric Disorders Checklist (TAND-SQ)
Source: Orphanet J Rare Dis. 2025 Jun 13;20:304. doi: 10.1186/s13023-025-03642-2 (PMC12166616; doi:10.1186/s13023-025-03642-2)
Supplement: Supplementary file 1 — Additional file 1. [file 13023_2025_3642_MOESM1_ESM.docx]

**Supplemental Material**

**Title:**

**Validation of the Self-Report Quantified Tuberous Sclerosis Complex-Associated Neuropsychiatric Disorders Checklist (TAND-SQ)**

**Authors:**

Nola Chambers, Tosca-Marie Heunis, Sugnet Gardner-Lubbe, Jamie K Capal, Stacey Bissell, Anna W Byars, Sebastián Cukier, Peter E Davis, Jennifer Flinn, Tanjala T Gipson, J Chris Kingswood, Aubrey J Kumm, Eva Schoeters, Catherine Smith, Shoba Srivastava, Megumi Takei, Stephanie Vanclooster, Agnies M van Eeghen, Robert Waltereit, Darcy A Krueger, Mustafa Sahin, Liesbeth De Waele, Anna C Jansen & Petrus J de Vries

**Internal consistency**

In our preliminary analyses, internal consistency was calculated for Yes/No responses (i.e., the cluster scores, CS) for only the *original* TAND-L items that formed the basis of the seven natural TAND clusters in earlier TAND cluster analysis studies (1–4), as well as for *all items* included in the new TAND-SQ (5). These analyses were only different for the autism-like cluster (with one new item), the neuropsychological cluster (with three new items), and the psychosocial clusters for the individual with TSC (with four new items) and caregiver (with seven new items). The results are presented in Supplemental Table 1. We noted that Cronbach’s alpha ($\alpha$) was higher for the autism-like cluster, neuropsychological cluster and psychosocial cluster for individuals with TSC when all the new items of the TAND-SQ were included. We therefore used all items in our subsequent cluster scores (CS) and cluster severity scores (CSS_mean_) for the validation analyses.

**Supplemental Table 1. Internal consistency of TAND-SQ cluster scores (CS)**

|  | **Cronbach’s Alpha (**$\boldsymbol{\alpha}$**)*** | |
| --- | --- | --- |
| **TAND Cluster** | **Including original TAND-L items** | **Including all new TAND-SQ items** |
| Autism-like | .79 (6 items) | .82 (7 items) |
| Dysregulated | .67 (3 items) | .67 (3 items) |
| Eat/Sleep | .37 (2 items) | .37 (2 items) |
| Mood/Anxiety | .67 (4 items) | .67 (4 items) |
| Neuropsychological | .84 (7 items) | .89 (10 items) |
| Overactive/Impulsive | .67 (3 items) | .67 (3 items) |
| Scholastic | .81 (4 items) | .81 (4 items) |
| Psychosocial-Individual  Psychosocial-Caregiver | .57 (3 items)  - | .78 (7 items)  .82 (7 items) |

* $\alpha$ values of 0.70 and above are considered acceptable.

**Discriminant validity**

Discriminant validity refers to the extent to which a test is not related to other tests that measure different constructs (6). We briefly looked at this aspect in our analyses of relationships between the TAND-SQ CS, CSS_mean_ and clinical diagnoses reported within the TAND-SQ in question 4. The expected relevant relationships were reported in Table 6 in the main paper. Here in Supplemental Table 2 we expand the information contained in Table 6 of the main paper to include preliminary analyses of discriminant validity of the TAND-SQ CS and CSS_mean_. Results show that nearly all CS and CSS_mean_ were significantly related to a self-reported diagnosis of autism, not just the autism-like CS and CSS_mean_. In contrast, an ADHD diagnosis was only significantly related to the Overactive/Impulsive cluster. Both Anxiety disorder and Depressive disorder were significantly associated with the mood/anxiety CS and CSS_mean_ as well as the autism-like CS and CSSmean (for anxiety disorder) and the scholastic CS (for Depressive disorder). The psychosocial CS for individuals with TSC were significantly associated with three of the four diagnoses (autism, anxiety disorder and depressive disorder), while the psychosocial CS for caregivers was associated with an autism diagnosis only. As mentioned in the main paper, a CSSmean score was not calculated for the psychosocial clusters as these items were not given a severity rating. The TTSS was significantly associated with a diagnosis of autism with a medium effect size and Anxiety disorder, with a small effect size.

**Supplemental Table 2.** **Spearman correlations (**$\boldsymbol{\rho}$**) between TAND CS, CSS_mean_ and TTSS, and clinical diagnoses as self-reported in the TAND-SQ for the total group (n=92)**

|  |  | **Diagnosis reported in question 4 of the TAND-SQ** | | | |
| --- | --- | --- | --- | --- | --- |
| **TAND Cluster** | **TAND Score** | **Autism**  ρ (p-value) | **ADHD**  ρ (p-value) | **Anxiety disorder**  ρ (p-value) | **Depressive disorder**  ρ (p-value) |
| Autism-like | CS | **.75 (p<.001)** | .16 (p=.121) | **.25 (p=.015)** | .01 (p=.896) |
|  | CSS_mean_ | **.75 (p<.001)** | .13 (p=.213) | **.21 (p=.049)** | .04 (p=.665) |
| Dysregulated | CS | **.47 (p<.001)** | .05 (p=.616) | .08 (p=.468) | -.07 (p=.482) |
|  | CSS_mean_ | **.48 (p<.001)** | .03 (p=.783) | -.09 (p=.400) | **-.24 (p=.021)** |
| Mood/Anxiety | CS | .06 (p=.542) | .01 (p=903) | **.41 (p<.001)** | **.29 (p=.005)** |
|  | CSS_mean_ | **.23 (p=.027)** | .11 (p=.282) | **.54 (p<.001)** | **.33 (p=.001)** |
| Neuropsychological | CS | **.65 (p<.001)** | .12 (p=.248) | .12 (p=.257) | -.01 (.957) |
|  | CSS_mean_ | **.56 (p<.001)** | .19 (p=.075) | .08 (p=479) | -.02 (p=.842) |
| Overactive/Impulsive | CS | **.46 (p<.001)** | .09 (p=405) | .13 (p=.229) | .01 (p=.952) |
|  | CSS_mean_ | **.43 (p<.001)** | **.28 (p=.007)** | .19 (p=.075) | .07 (p=.494) |
| Scholastic | CS | **.54 (p<.001)** | .05 (p=.686) | .03 (p=.830) | **-.25 (p=.028)** |
|  | CSS_mean_ | **.46 (p<.001)** | .13 (p=.206) | .16 (p=.125) | -.10 (p=.335) |
| Psychosocial-Individual | CS | **.31 (p=.002)** | .05 (p=.631) | **.24 (p=.023)** | **.26 (p=.013)** |
| Psychosocial-Caregiver (n=66) | CS | **.38 (p=.001)** | .03 (p=.814) | .23 (p=.059) | .02 (p=.885) |
| TTSS_mean_ | TTSS_mean_ | **.69 (p<.001)** | .17 (p=.107) | **.21 (p=.047)** | .01 (p=.966) |

Spearman correlations ($\rho$) with p-values of <.05 were considered significant and are in bold.

These results suggest that the CS and CSS_mean_ for the mood-anxiety cluster and the overactive/impulsive clusters show some discriminant validity with respect to self-reported clinical diagnoses. The fact that all the cluster CS and CSS_mean_ correlated with a diagnosis of autism suggests high overlap in symptom profiles of autism and a wide range of TAND behaviours. This finding could also suggest a high degree of undiagnosed comorbidities in those diagnosed with autism, and overlapping profiles of symptoms across diagnostic categories (7,8). These results are too limited for true interpretation, especially considering these were self-report diagnoses, but were included here as support for further research into discriminant validity of the TAND-SQ.

**References:**

1. Alperin S, Krueger DA, Franz DN, Agricola KD, Stires G, Horn PS, et al. Symptom rates and profile clustering in tuberous sclerosis complex-associated neuropsychiatric disorders (TAND). J Neurodev Disord. 2021;13:60.

2. de Vries PJ, Leclezio L, Gardner-Lubbe S, Krueger D, Sahin M, Sparagana S, et al. Multivariate data analysis identifies natural clusters of Tuberous Sclerosis Complex Associated Neuropsychiatric Disorders (TAND). Orphanet J Rare Dis. 2021;16:447.

3. Leclezio L, Gardner-Lubbe S, de Vries PJ. Is It Feasible to Identify Natural Clusters of TSC-Associated Neuropsychiatric Disorders (TAND)? Pediatr Neurol. 2018;81:38–44.

4. de Vries PJ, Belousova E, Benedik MP, Carter T, Cottin V, Curatolo P, et al. Natural clusters of tuberous sclerosis complex (TSC) -associated neuropsychiatric disorders (TAND): new findings from the TOSCA TAND research project. J Neurodev Disord. 2020;12:24.

5. Heunis TM, Chambers N, Vanclooster S, Bissell S, Byars AW, Capal JK, et al. Development and feasibility of the self-report, quantified TSC-Associated Neuropsychiatric Disorders Checklist (TAND-SQ). Pediatr Neurol. 2023;147:101–23.

6. Flake JK, Pek J, Hehman E. Construct Validation in Social and Personality Research: Current Practice and Recommendations. Soc Psychol Personal Sci. 2017;8(4):370–8.

7. Colvert E, Simonoff E, Capp SJ, Ronald A, Bolton P, Happé F. Autism Spectrum Disorder and Mental Health Problems: Patterns of Difficulties and Longitudinal Trajectories in a Population-Based Twin Sample. J Autism Dev Disord. 2022;52(3):1077–91.

8. Coughlan B, Woolgar M, Van Ijzendoorn MH, Duschinsky R. Socioemotional profiles of autism spectrum disorders, attention deficit hyperactivity disorder, and disinhibited and reactive attachment disorders: A symptom comparison and network approach. Dev Psychopathol. 2023;35:1026–35.
